# Effect of Mediterranean Diet and Antioxidant Formulation in Non-Alcoholic Fatty Liver Disease: A Randomized Study

**DOI:** 10.3390/nu9080870

**Published:** 2017-08-12

**Authors:** Ludovico Abenavoli, Marta Greco, Natasa Milic, Francesca Accattato, Daniela Foti, Elio Gulletta, Francesco Luzza

**Affiliations:** 1 Department of Health Sciences, University “Magna Græcia”, 88100 Catanzaro, Italy; marta.greco@unicz.it (M.G.); francescaaccattato@libero.it (F.A.); foti@unicz.it (D.F.); gulletta@unicz.it (E.G.); luzza@unicz.it (F.L.); 2 Department of Pharmacy, University of Novi Sad, 21000 Novi Sad, Serbia; milnat@libero.it

**Keywords:** non-alcoholic fatty liver disease, Mediterranean diet, insulin resistance, weight, antioxidant

## Abstract

Non-alcoholic fatty liver disease (NAFLD) is the most common liver disease worldwide, characterized by liver fatty acid accumulation and fibrosis, not due to excessive alcohol consumption. Notably, nutritional habits have been reported to be implicated in the onset and severity of the hepatic damage, while the Mediterranean diet has shown beneficial effects on NAFLD. Free radicals and oxidative stress were suggested to be involved in the pathogenesis and progression of NAFLD, and several data highlighted the efficacy of antioxidant supplementation in its treatment. The aim of this study was to compare the effects of the Mediterranean diet, with or without an antioxidant complex supplement, in overweight patients suffering from NAFLD. In this prospective study, fifty Caucasian overweight patients were randomized into three groups (Groups A–C). A personalized moderately hypocaloric Mediterranean diet was prescribed to all patients included in the A and B groups. In addition to the diet, Group B was administered antioxidant supplementation daily and for the period of six months. Group C did not have any type of treatment. The study proved that the Mediterranean diet alone or in association with the antioxidant complex improved anthropometric parameters, lipid profile and reduced hepatic fat accumulation and liver stiffness. However, Group B patients, in which the diet was associated with antioxidant intake, showed not only a significant improvement in insulin sensitivity, but also a more consistent reduction of anthropometric parameters when compared with Group A patients. Taken together, these results support the benefit of antioxidant supplementation in overweight patients with NAFLD.

## 1. Introduction

Non-alcoholic fatty liver disease (NAFLD) is an emerging public health issue, being not only a major cause of liver-related morbidity and mortality worldwide, but also an independent risk factor for cardiovascular diseases [1,2,3]. It refers to a wide spectrum of liver injuries, ranging from steatosis to non-alcoholic steatohepatitis (NASH), fibrosis, cirrhosis and related complications [4,5]. The Dionysos Nutrition and Liver Study and the Dallas Heart Study have calculated that 25% of Italian and 30% of American citizens are affected by NAFLD [6,7].

NAFLD is now considered the hepatic border of metabolic syndrome, supported by a high-calorie dietetic regimen, in the presence of a genetic profile characterized by predisposing polymorphisms [4,8,9]. Several pathogenic hypotheses on NAFLD development have been formulated. Both the early “two-hit” model and the more recent “multi parallel hit” hypothesis suggest that fat accumulation in the hepatocytes exposes the liver to oxidative stress with reactive oxygen species (ROS) production, inflammation unbalance, cellular necrosis and, finally, fibrosis [10,11,12,13,14].

In particular, several pieces of evidence both in animal models and in humans support a key role of oxidative stress in the progression of NAFLD [13,15]. An increase in liver and serum content of oxidation products and a decrease in plasma antioxidant activity has been documented in NAFLD patients [16,17]. It has also been demonstrated that ROS production is greater in patients with NASH compared with those with simple steatosis [12,18,19], and further studies showed improvement of NASH after taking antioxidant supplements, indirectly supporting an involvement of oxidative stress in the progression of steatosis [20,21,22].

High-calorie diet represents an underlying mechanism favoring ROS generation and, consequently, increased requirements of antioxidant enzymes [14], such as glutathione reductase, which increases to limit the oxidative damage, particularly in severely obese individuals [15]. NAFLD is frequently asymptomatic and accidentally discovered by blood liver function tests or instrumental investigations.

NAFLD treatment entails a consistent change in lifestyle habits. Literature data and international guidelines have highlighted the health benefits linked to weight loss and physical exercise [23]. In this way, the Mediterranean diet appears to be a perfect fit for NAFLD patients, due to its efficacy on liver status leading to improvement of insulin sensitivity and lipid profile, but also for being a primary form of prevention for NAFLD-related diseases [24,25,26].

However, there is currently no consensus regarding the pharmacological treatment of NAFLD. Many drugs have been tested and proposed to prevent or improve NAFLD. The most common approach has probably been the use of classical oral anti-diabetic medications including thiazolidinediones, whose use combines insulin-sensitizing and anti-proliferative effects [27,28], as well as lipid lowering agents [23].

Recently, we have described a series of cases involving overweight patients suffering from NAFLD, for which lifestyle changes associated with the administration of a new antioxidant complex supplement, recently introduced in the Italian market (Bilirel (BIL), Pharmaluce, San Marino, Republic of San Marino), have led to decreased liver fat accumulation and weight reduction [29]. The composition of one pill of BIL is silymarin 120 mg, chlorogenic acid 7.5 mg, protopine 0.04 mg, l-methionine 150 mg and l-glutathione 10 mg.

This randomized study aims to evaluate the effects of the BIL complex associated with Mediterranean diet on liver fat accumulation, glucose and lipid metabolism and on anthropometric parameters in NAFLD overweight patients.

## 2. Patients and Methods

In this prospective study, 50 Caucasian overweight patients with body mass index (BMI) greater than 25 kg/m^2^, who regularly attended our outpatient Gastroenterology clinic, were consecutively enrolled from June 2015–June 2016. Our study protocol is consistent with the ethical guidelines of the Helsinki 1975 Declaration and was approved by the local Ethical Committee (Comitato etico regionale, Sezione Area Centro, Catanzaro, Italy). Informed Consent was obtained for each patient enrolled in the study. Patients affected by hepatitis B and C, with cardiac, renal, autoimmune and metabolic diseases were excluded from the study. Exclusion criteria were also insulin treatment, smoking habits, alcohol intake (>20 g/day), recreational drug use and exposure to environmental toxins known to induce liver steatosis.

Patients were randomized by a systematic sampling procedure into three groups (Groups A–C). A personalized moderately low-calorie Mediterranean diet (1400–1600 kcal/day) was conceived of and prescribed to Group A and B patients for six months. Diet composition was designed to get an animal to vegetable protein ratio of 1:1. The Italian Recommended Dietary Allowances (RDAs) were incorporated to ensure proper vitamin and mineral intake [30,31]. The proposed food plan included carbohydrates (50–60%), proteins (15–20%, about 50% of which were vegetable proteins), mono- and poly-unsaturated fats (less than 30%), saturated fat (less than 10%), cholesterol (less than 300 mg/day) and fibers (25–30 g/day).

In association with the diet, Group B patients were administered two pills of BIL complex daily, for six months. This dose of BIL complex is consistent with our previously published data and it is currently used in clinical practice [29]. Daily physical activity was strongly recommended for patients of both Groups A and B [15,32]. Strict observation of dietary and physical activity prescriptions was monitored through a monthly phone interview. Finally, patients of the control group (Group C), who had not undertaken any pharmacological treatment and lifestyle changes, were monitored for the same period, with the medical indication to reduce body weight.

### 2.1. Clinical Parameters and Blood Sample Collection

Anthropometric parameters (body weight, height, waist and hip circumferences), systolic and diastolic blood pressure were assessed according to standard methods. BMI was calculated as body weight divided by height squared (kg/m^2^). Blood was collected, after 12–14 h from fasting, by an antecubital venous puncture. Serum or plasma samples were obtained by centrifugation. Serum levels of total cholesterol, low density cholesterol (LDL-C), triglycerides (TG), fasting glucose, insulin, creatinine, urea and standard liver tests, including total bilirubin, aspartate aminotransferase (AST), alanine aminotransferase (ALT) and gamma-glutamyl transpeptidase (γGT), were directly measured using standard automated laboratory methods on Cobas 6000 (Roche, Rotkreuz, Switzerland), by using the relative kits, according to the manufacturer’s instructions. To evaluate insulin resistance, we calculated two surrogate indexes: the homeostasis model assessment of insulin resistance (HOMA-IR) and the product of the plasma triglyceride and glucose concentrations (TyG) index [33,34].

### 2.2. Steatosis Evaluation

To diagnose NAFLD, liver ultrasound (US) examination was performed. The hepatic fat accumulation grade was calculated as follows: absent (score 0), mild (score 1), moderate (score 2) and severe (score 3), according to the Hamaguchi score, which uses a 6-point scoring system based on hepatorenal echo contrast, liver brightness, deep attenuation and vascular blurring. This scoring system showed 100% specificity and 91.7% sensitivity when compared with liver biopsy [35].

In line with the recent development of new non-invasive methods to detect the presence of liver steatosis, we used also the fatty liver (FL) index, a validated algorithm based on BMI, waist circumference, TG and γGT values, which ranges from 0–100, with an accuracy of 0.84 (95% confidence interval (CI) 0.81–0.87) [36]. The FL index is thus calculated as:

FL index = (e^0.953*log^_e_^(triglycerides) + 0.139*BMI + 0.718*log^_e_^(γGT) + 0.053*waist circumference - 15.745^)/(1 + e^0.953*log^_e_^(triglycerides) + 0.139*BMI + 0.718*log^_e_^(γGT) + 0.053*waist circumference − 15.745^) × 100 (1)

An FL index <30 (negative likelihood ratio = 0.2) rules out steatosis, whereas an FL index ≥60 (positive likelihood ratio = 4.3) identifies steatosis.

### 2.3. Liver Fibrosis Assessment

Each patient underwent liver stiffness measurement by transient elastography (TE) (FibroScan^®^, Echosens, Paris, France) using the M probe. This medical device provides a quantifiable estimate of liver stiffness in kilopascal (kPa); measurements were considered representative only if they scored at least 10 valid acquisitions with a success rate >60% [37,38]. All measurements were taken by the same operator (experience >10,000 measurements) who was unaware of other patient’s parameters.

## 3. Data Analysis

Statistical analyses were performed by SPSS 20.0 software for Windows. Each analyte was tested for normality using the Kolmogorov–Smirnov test. Because of the non-normal distribution of the studied variables, they were indicated as the median and interquartile range (IQR).

The non-parametric Wilcoxon test was used to evaluate the intragroup differences between the anthropometric variables and laboratory parameters at zero time and after six months. To evaluate the intergroup percentage changes in all traits during the time of the study, the non-parametric Mann–Whitney test was performed. The percentage variations from baseline to six months were calculated as ∆T0/T6 = (T6 − T0) / T0 × 100. A *p*-value less than 0.05 was regarded as statistically significant.

## 4. Results

Clinical, anthropometric and laboratory parameters of all patients stratified in the three groups are reported in Table 1. Patients were subdivided using a systematic random sampling procedure: Group A included 20 patients (12♂ and 8♀; median age 52 (IQR: 40–60) years); Group B included 20 patients (16♂ and 4♀; median age 46 (IQR: 40–57) years); Group C included 10 patients (6♂ and 4♀; median age 33 (IQR: 28–53) years). We did not report side effects or dropouts during the study, and full adherence to the study protocol was registered. Compliance with the study protocol was evaluated through a weekly phone interview for Group A and B patients.

Group A patients showed after six months a statistically-significant decrease in weight, BMI, waist and hip circumference (*p* = 0.0001) and an improvement of γGT (*p* = 0.024), lipid profile with a significant decrease in TG (*p* = 0.0001), total cholesterol (*p* = 0.0001) and LDL-C (*p* = 0.005). A significant decrease was also detected in the FL index (*p* = 0.002) and TE values (*p* = 0.0001) (Table 2).

In Group B patients, a statistically-significant decrease after six months of treatment was observed in body weight (*p* = 0.02), BMI (*p* = 0.0001), waist (*p* = 0.0001) and hip circumference (*p* = 0.001), systolic blood pressure (*p* = 0.012), as well as in ALT (*p* = 0.007), fasting glucose (*p* = 0.007), insulin (*p* = 0.0001), TG (*p* = 0.011), total cholesterol (*p* = 0.0001) and LDL-C (*p* = 0.016) blood levels.

Moreover, in Group B, we observed not only a significant decrease in the FL index (*p* = 0.003) and TE values (*p* = 0.0001), but also in both insulin resistance indexes, HOMA-IR (*p* = 0.001) and TyG (*p* = 0.005) (Table 3). Conversely, in the control group (C), statistically-significant improvements in AST (*p* = 0.023), γGT (*p* = 0.036), insulin (*p* = 0.041), total cholesterol (*p* = 0.025) and LDL-C (*p* = 0.028) blood levels were reported after six months (Table 4). Group C patients even showed an increase in the HOMA-IR index (*p* = 0.024).

Intergroup percentage changes in all traits (ΔT0/T6) from baseline (T0) to six months (T6) were evaluated by the Mann-Whitney test (Table 5). The analysis between Groups A and C showed statistically-significant variations in anthropometric parameters, such as weight and BMI (*p* = 0.0001), waist (*p* = 0.0001), hip circumference (*p* = 0.001), lipid profile: TG (*p* = 0.001) and total cholesterol (*p* = 0.0001), insulin sensitivity: insulin (*p* = 0.045), HOMA-IR (*p* = 0.021), TyG index (*p* = 0.020), but also in the FL index (*p* = 0.017) and TE values (*p* = 0.001).

A comparison between Groups B and C showed substantially the same differences in anthropometric parameters described above (weight (*p* = 0.030), BMI (*p* = 0.0001), waist (*p* = 0.0001) and hip circumference (*p* = 0.001)). A significant improvement in glycemic metabolism (insulin (*p* = 0.0001), fasting glucose levels (*p* = 0.006), HOMA-IR (*p* = 0.001), TyG index (*p* = 0.01)), in steatosis evaluation by the FL index (*p* = 0.0001) and TE values (*p* = 0.0001) has also been observed.

**Figure 1:**
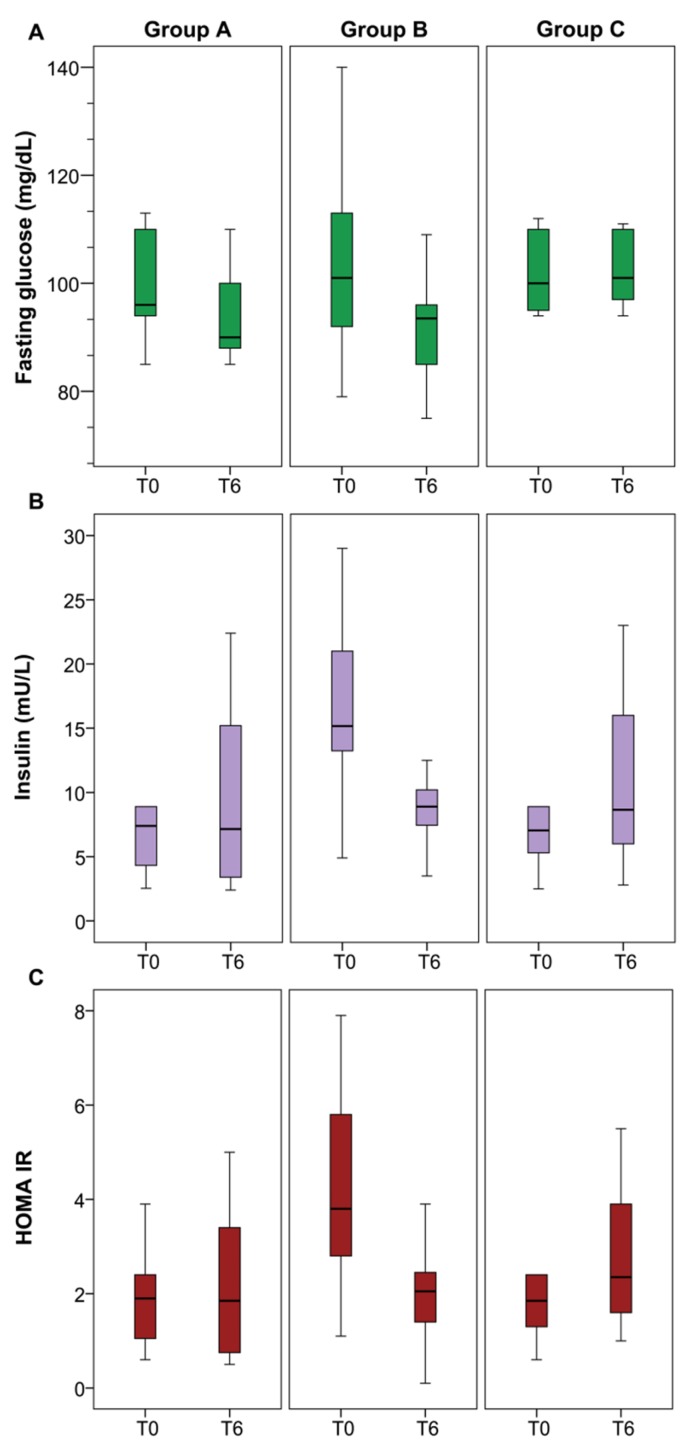
Comparison of fasting glucose (**A**); insulin (**B**) and HOMA-IR (**C**) at baseline (T0) and after six months (T6), among the three groups (Group A = diet; Group B = diet/BIL complex; Group C = control, without diet and antioxidant supplementation).

However, Group B patients, who underwent both diet and BIL complex treatment, showed a statistically-significant reduction in fasting glucose (*p* = 0.016), insulin levels (*p* = 0.0001) and, consequently, HOMA-IR index (*p* = 0.0001), as compared to Group A patients (Figure 1). Finally, inter-group analysis of all tested parameters showed that fasting glucose and insulin level, HOMA-IR, FL index and TE values were significantly lower in Group B than in Group A, after the treatment period (Table 5).

## 5. Discussion

Despite the rapidly growing recognition of NAFLD over the last few decades, the treatment of this condition remains debated [39,40]. In the clinical management of NAFLD patients, a dietary change and increased physical exercise are essential to reduce body weight, in order to improve metabolic parameters and normalize the biochemical blood profile, as well as transaminase levels [24]. The “ideal” treatment for NAFLD should reduce the liver damage and its progression by reducing anthropometric parameters, by improving insulin resistance and impairment in glucose and lipid metabolism and by reducing the cytokine-mediated pathophysiological link between adipose tissue and liver [41]. The traditional Mediterranean diet is a dietary pattern that was associated with favorable health impact, in particular on cardiovascular diseases, cancer and in the treatment of metabolic syndromes [42]. Carotenoids, fibers and folic acid, which are basic components of this diet, can play a pivotal role in preventing or slowing down the oxidative stress process. In addition, vegetables, which are the staple foods included in the Mediterranean diet, are the main source of phytosterols, known as natural cholesterol-lowering agents, reducing cardiovascular risk [43,44].

Several pharmaceutical agents are currently being evaluated for the treatment of NAFLD, and NASH in particular. However, no single therapy has been approved so far [23,45]. On this basis, the beneficial effects of complementary medicine, and particularly of herbal extracts, on NAFLD patients have received increasing attention in the last few years. The use of this approach has many advantages, including worldwide availability, minimal reported side effects and wide application due to low treatment costs [46].

However, literature data are often inconclusive on this topic, due to the high number of biases found in many trials and to the limited number of studies testing single herbal remedies [47].

In the last two decades, several studies have emphasized the benefits in the NAFLD treatment of *Silybum marianum*, commonly called milk thistle (MT), a plant native to the Mediterranean area, which has been used for many centuries to treat liver diseases [48,49]. The active complex of MT is a lipophilic extract from the seeds of the plant, and it is composed by three flavonolignan isomers, silybin, silydianin and silychristin, collectively called silymarin.

Studies of patients with NAFLD showed that silymarin treatment was associated with positive changes in insulin resistance and transaminase serum levels [50,51]. Loguercio et al., in a multicenter phase III double-blind clinical trial, showed that MT extracts, after 12 months, led to an improvement of insulin resistance, liver enzymes and liver histology, without any increase in body weight in NAFLD patients [52]. More recently, in a randomized clinical study, we have found out that Mediterranean diet, in association with silymarin and other antioxidants, is able to induce, after six months, significant changes in glucose and lipid metabolism [53].

According to these data, in our cohort, we demonstrated an improvement of BMI, waist and hip circumference, TG, total cholesterol and LDL-C serum level in all patients who followed the Mediterranean diet for a period of six months (Group A and B). The diet also led to the decrease of intra-hepatic fat accumulation, evaluated by the FL index, and of liver stiffness, assessed by TE. However, in the overweight NAFLD Group B patients, who followed the Mediterranean diet in association with BIL antioxidant treatment, we reported the statistical reduction of the HOMA-IR and the TyG index, two surrogate indexes widely used to evaluate insulin resistance.

The changes in glucose and lipid metabolism described in Group B can be explained also by the presence of chlorogenic acid, one component of the BIL complex. Chlorogenic acid is one of the most abundant polyphenols in the human diet. It is contained in coffee, fruits and vegetables and displays many biological properties, such as antidiabetic effects by stimulating glucose uptake in both insulin-sensitive and insulin-resistant adipocytes and by improving early fasting glucose and insulin responses [54]. The metabolic changes observed in our study can be explained by the synergic action of the Mediterranean diet in association with chlorogenic acid and silymarin.

Another component of the BIL complex is protopine, an isoquinoline alkaloid present in *Fumaria officinalis*, with antioxidant and choleretic properties that inhibit the production of pro-inflammatory cytokines [55]. Our data suggest that protopine could be a potential candidate for NAFLD treatment.

The increase in oxidative stress and free radical production observed in NAFLD lead not only to increased consumption of glutathione, the major intra-cellular antioxidant, but it also reduces the activity of s-adenosyl-l-methionine, the main biological methyl donor and a precursor of glutathione, essential for protecting antioxidant pathways [56]. Recent studies suggest that the reduction of glutathione levels, in combination with lower ATP availability due to mitochondrial deregulation, leads to an unbalance of reactive oxygen species production and to the subsequent progression of hepatic injury [57]. In this context, the administration of reduced glutathione and methionine can help to restore the oxidative balance.

The BIL antioxidant complex treatment alone, not in association with physical activity and a calorie-controlled diet, is not effective in improving insulin resistance. However, our data confirm the possible therapeutic role of this antioxidant complex as a complementary approach to the treatment of overweight NAFLD patients and in particular in the management of insulin resistance in NAFLD-related pathologies.

An important goal for modern hepatologists is to find effective non-invasive diagnostic approaches to NAFLD. In the last two decades, non-invasive diagnostic modalities for NAFLD have been investigated. On the basis of literature data, three non-invasive methods have been employed in the present study for the evaluation of NAFLD. In addition to the US examination, in particular, the FL index and TE have been used to assess respectively hepatic fat accumulation and liver stiffness. The FL index is an accurate and easy to employ predictor score to define steatosis presence that utilizes routine measurements in clinical practice such as a BMI, waist circumference, triglycerides and γGT [36]. In this way, the clinical use of the FL index is useful to identify patients with NAFLD to include in an outpatient lifestyle change program. The data on the reduction of hepatic fat accumulation were also confirmed by the reduction of the Hamaguchi score at the US examination in Groups A and B, compared to Group C.

TE is a non-invasive tool for the evaluation of liver damage that demonstrated good accuracy in quantifying the levels of hepatic stiffness and to define fibrosis, in patients with liver diseases and in particular with NAFLD [37]. This technique is reliable, fast and reproducible, with a good intra- and inter-observer agreement, thus allowing for population-wide screening and disease follow-up.

Finally, our study clearly shows that patients following a balanced diet and taking the antioxidant complex had a more significant attenuation of insulin resistance, hepatic fat accumulation and liver stiffness than patients following the diet alone. These results supported the effectiveness of the BIL complex to reduce liver fatty acid infiltration and its related damages, by positively influencing the mitochondrial function and by reducing oxidative stress.

## 6. Conclusions

Our study confirms that the Mediterranean diet can improve anthropometric parameters and lipid profile and can contribute to reducing hepatic fat accumulation and liver stiffness. Moreover, the association of this dietetic regimen with antioxidant supplementation can contribute to improving the insulin sensitivity parameters. These data support a possible role of antioxidant supplementation as a coadjuvant therapy in patients with NAFLD.

## Figures and Tables

**Table 1:** Characteristics of the study population stratified into the three groups, at baseline.

	Group A (*n* = 20)	Group B (*n* = 20)	Group C (*n* = 10)
Age (years)	52 (40–60)	46 (40–57)	33 (28–43)
Weight (kg)	83 (80–88)	90 (81–92)	84 (75–95)
BMI (kg/m^2^)	31 (29–33)	29 (28–32)	29 (27–31)
Waist circumference (cm)	108 (99–114)	104 (100–105)	102 (97–110)
Hip circumference (cm)	105 (102–116)	105 (102–110)	105 (99–112)
Systolic blood pressure (mmHg)	125 (120–140)	130 (120–140)	120 (110–130)
Diastolic blood pressure (mmHg)	80 (70–90)	80 (70–90)	80 (60–82)
AST (U/L)	22 (20–25)	22 (20–25)	25 (23–35)
ALT (U/L)	22 (15–30)	25 (21–40)	35 (24–58)
γGT (U/L)	20 (16–28)	21 (14–31)	21 (16–37)
Total bilirubin (mg/dL)	0.45 (0.38–0.80)	0.40 (0.30–0.60)	0.80 (0.77–0.90)
Fasting glucose (mg/dL)	96 (94–110)	101 (90–113)	100 (95–110)
Insulin (mU/L)	8 (4–16)	15 (13–21)	7 (5–11)
Triglycerides (mg/dL)	140 (129–157)	101 (84–106)	138 (115–173)
Total cholesterol (mg/dL)	189 (178–206)	198 (171–213)	176 (147–191)
LDL-C (mg/dL)	124 (105–134)	122 (97–133)	115 (99–125)
Creatinine (mg/dL)	0.8 (0.7–0.8)	0.8 (0.7–0.9)	0.80 (0.70–0.90)
Urea (mg/dL)	38 (31–43)	38 (28–40)	32 (26–40)
HOMA-IR	2 (1–2)	4 (3–6)	2 (1–3)
TyG index	4.7 (4.7–4.8)	4.6 (4.5–4.8)	4.7 (4.7–4.8)
FL index	71 (56–85)	58 (42–69)	67 (63–80)
TE	8.1 (6.7–9.2)	6.9 (6.7–7.2)	7.2 (5.3–10.1)
US score	2 (2–3)	2 (2–2)	1 (0.75–2)

Continuous variables are expressed as median and quartiles (IQR). BMI: body mass index; AST: aspartate aminotransferase; ALT: alanine aminotransferase; γGT: γ-glutamyl transpeptidase; LDL: low-density lipoprotein cholesterol; HOMA-IR: homeostasis model assessment-insulin resistance; TyG index: triglyceride-glucose index; FL index: fatty liver index; TE: transient elastography; US score: ultrasound score.

**Table 2:** Comparison of anthropometric, biochemical and clinical features at baseline (T0) and after six months (T6) of treatment in patients following a low-calorie diet (Group A).

	Group A		
	T0	T6	*p*
Weight (kg)	83 (80–88)	78 (75–80)	0.0001
BMI (kg/m^2^)	31 (29–33)	29 (27–31)	0.0001
Waist circumference (cm)	108 (99–114)	102 (98–111)	0.0001
Hip circumference (cm)	105 (102–116)	102 (99–110)	0.0001
Systolic blood pressure (mmHg)	125 (120–140)	125 (120–130)	0.121
Diastolic blood pressure (mmHg)	80 (70–90)	80 (70–80)	0.755
AST (U/L)	22 (20–25)	23 (21–25)	0.101
ALT (U/L)	22 (15–30)	25 (18–31)	0.497
γGT (U/L)	20 (16–28)	25 (21–31)	0.024
Total bilirubin (mg/dL)	0.45 (0.38–0.80)	0.45 (0.30–0.80)	0.436
Fasting glucose (mg/dL)	96 (94–110)	90 (88–102)	0.258
Insulin (mU/L)	8 (4–16)	7 (3–15)	0.777
Triglycerides (mg/dL)	140 (129–157)	85 (75–135)	0.0001
Total cholesterol (mg/dL)	189 (178–206)	156(143–185)	0.0001
LDL-C (mg/dL)	124 (105–134)	102 (92–115)	0.005
Creatinine (mg/dL)	0.8 (0.7–0.8)	0.8 (0.7–0.9)	0.218
Urea (mg/dL)	38 (31–43)	30 (27–34)	0.007
HOMA-IR	1.9 (0.9–2.4)	1.8 (0.6–3.4)	0.985
TyG index	4.7 (4.7–4.8)	4.5 (4.4–4.8)	0.100
FL index	71 (56–85)	45 (39–69)	0.002
TE	8.1 (6.7–9.2)	6.0 (5.1–7.0)	0.0001
US score	2 (2–3)	2 (1–2)	0.0001

Non-parametric Wilcoxon test. A *p*-value less than 0.05 is considered statistically significant. BMI: body mass index; AST: aspartate aminotransferase; ALT: alanine aminotransferase; γGT: γ-glutamyl transpeptidase; LDL: low-density lipoprotein cholesterol; HOMA-IR: homeostasis model assessment-insulin resistance; TyG index: triglyceride-glucose index; FL index: fatty liver index; TE: transient elastography; US score: ultrasound score.

**Table 3:** Comparison of anthropometric, biochemical and clinical features at baseline (T0) and after six months (T6) of treatment in patients following a low-calorie diet in association with the Bilirel (BIL) complex (Group B).

	Group B		
	T0	T6	*p*
Weight (kg)	90 (81–92)	81 (74–86)	0.002
BMI (kg/m^2^)	29 (28–32)	27 (25–28)	0.0001
Waist circumference (cm)	104 (100–105)	98 (96–100)	0.0001
Hip circumference (cm)	105 (102–110)	101 (99–102)	0.001
Diastolic blood pressure (mmHg)	80 (70–90)	80 (70–80)	0.285
Systolic blood pressure (mmHg)	130 (120–140)	120 (120–130)	0.012
AST (U/L)	22 (20–25)	21 (18–32)	0.955
ALT (U/L)	25 (21–40)	25 (17–25)	0.007
γGT (U/L)	21 (14–31)	24 (16–30)	0.175
Total bilirubin (mg/dL)	0.40 (0.30–0.60)	0.50 (0.40–0.60)	0.084
Fasting glucose (mg/dL)	101 (90–113)	93 (85–96)	0.007
Insulin (mU/L)	15 (13–21)	9 (7–10)	0.0001
Triglycerides (mg/dL)	106 (100–139)	75 (61–92)	0.011
Total cholesterol (mg/dL)	198 (171–213)	152 (140–180)	0.0001
LDL-C (mg/dL)	122 (97–133)	98 (78–120)	0.016
Creatinine (mg/dL)	0.8 (0.7–0.9)	95 (78–120)	0.409
Urea (mg/dL)	38 (28–40)	39 (28–44)	0.497
HOMA-IR	4 (3–6)	2 (1–2)	0.001
TyG index	4.6 (4.5–4.8)	4.5 (4.5–4.7)	0.005
FL index	58 (42–69)	38 (29–45)	0.003
TE	6.9 (6.7–7.2)	5.0 (4.7–5.2)	0.0001
US score	2 (2–2)	0 (0–1)	0.0001

Non-parametric Wilcoxon test. A *p*-value less than 0.05 would be considered statistically significant. BMI: body mass index; AST: aspartate aminotransferase; ALT: alanine aminotransferase; γGT: γ-glutamyl transpeptidase; LDL: low-density lipoprotein cholesterol; HOMA-IR: homeostasis model assessment-insulin resistance; TyG index: triglyceride-glucose index; FL index: fatty liver index; TE: transient elastography; US score: ultrasound score.

**Table 4:** Comparison of anthropometric, biochemical and clinical features at baseline (T0) and after six months (T6) in control patients (Group C).

	Group C		
	T0	T6	*p*
Weight (kg)	84 (75–95)	85 (75–95)	0.214
BMI (kg/m^2^)	29 (27–31)	29 (27–30)	0.223
Waist circumference (cm)	102 (97–110)	102 (99–111)	0.334
Hip circumference (cm)	105 (99–112)	106 (99–113)	0.389
Systolic Blood Pressure (mmHg)	120 (110–130)	120 (120–132)	0.066
Diastolic Blood Pressure (mmHg)	80 (60–82)	80 (70–82)	0.102
AST (U/L)	25 (23–35)	29 (24–56)	0.023
ALT (U/L)	35 (24–58)	40 (32–52)	0.878
γGT (U/L)	21 (16–37)	29 (21–37)	0.036
Total Bilirubin (mg/dL)	0.80 (0.77–0.90)	0.80 (0.75–0.90)	0.257
Fasting Glucose (mg/dL)	100 (95–110)	101 (96–110)	0.395
Insulin (mU/L)	7 (5–11)	9 (6–16)	0.041
Triglycerides (mg/dL)	138 (115–173)	143 (138–173)	0.241
Total Cholesterol (mg/dL)	176 (147–191)	152 (140–180)	0.025
LDL-C (mg/dL)	115 (99–125)	78 (54–95)	0.028
Creatinine (mg/dL)	0.80 (0.70–0.90)	0.8 (0.77–0.90)	0.083
Urea (mg/dL)	32 (26–40)	31 (28–36)	0.776
HOMA-IR	1.8 (1.2–2.8)	2.3 (1.6–3.9)	0.024
TyG index	4.7 (4.7–4.8)	4.8 (4.8–4.9)	0.132
FL index	67 (63–80)	69 (68–83)	0.066
Fibroscan	7.2 (5.3–10.1)	8.5 (6.3–9.7)	0.683
US score	1 (0.75–2)	1 (1–2)	0.705

Non-parametric Wilcoxon test. A *p*-value less than 0.05 is considered statistically significant. BMI: body mass index; AST: aspartate aminotransferase; ALT: alanine aminotransferase; γGT: γ-glutamyl transpeptidase; LDL: low-density lipoprotein cholesterol; HOMA-IR: homeostasis model assessment-insulin resistance; TyG index: triglyceride-glucose index; FL index: fatty liver index; TE: transient elastography; US score: ultrasound score.

**Table 5:** Effects of diet and diet/BIL complex association on anthropometric, clinical and biochemical parameters.

	Group A	Group B	Group C	*p*
Weight (kg)	6% (−)	7% (−)	0.5% (−)	A vs. C **0.0001**
				B vs. C **0.030**
				A vs. B 0.665
BMI (kg/m^2^)	7.5% (−)	9% (−)	0.45% (−)	A vs. C **0.0001**
				B vs. C **0.0001**
				A vs. B 0.935
Waist circumference (cm)	2.8% (−)	6% (−)	0.3% (−)	A vs. C **0.0001**
				B vs. C **0.0001**
				A vs. B **0.030**
Hip circumference (cm)	3.3% (−)	4% (−)	0.7% (−)	A vs. C **0.001**
				B vs. C **0.001**
				A vs. B 0.206
Fasting glucose (mg/dL)	3.5% (−)	11% (−)	0.5% (−)	A vs. C 0.724
				B vs. C **0.006**
				A vs. B **0.016**
Insulin (mU/L)	10% (+)	38% (−)	25% (+)	A vs. C **0.045**
				B vs. C **0.0001**
				A vs. B **0.0001**
Triglycerides (mg/dL)	32.16% (−)	21% (−)	2.8% (+)	A vs. C **0.001**
				B vs. C **0.002**
				A vs. B 0.935
Total cholesterol (mg/dL)	14.8% (−)	17% (−)	9.3% (+)	A vs. C **0.0001**
				B vs. C **0.0001**
				A vs. B 0.626
LDL-C (mg/dL)	15% (−)	9% (−)	29% (−)	A vs. C 0.217
				B vs. C 0.234
				A vs. B 0.705
HOMA-IR	6.2% (+)	43% (−)	46% (+)	A vs. C **0.021**
				B vs. C **0.001**
				A vs. B **0.0001**
TyG index	3.3% (−)	1.2% (−)	1% (+)	A vs. C **0.020**
				B vs. C **0.010**
				A vs. B 0.131
FL index	19% (−)	27% (−)	4.7% (+)	A vs. C **0.017**
				B vs. C **0.0001**
				A vs. B 0.626
TE	21% (−)	27% (−)	8.7% (+)	A vs. C **0.001**
				B vs. C **0.0001**
				A vs. B 0.053

The non-parametric Mann–Whitney test was used to evaluate the intergroup percentage changes (*p* < 0.05); (−) reduction, (+) increase. BMI: body mass index; LDL: low-density lipoprotein cholesterol; HOMA-IR: homeostasis model assessment-insulin resistance; TyG index: triglyceride-glucose index; FL index: fatty liver index; TE: transient elastography.
